# Postnatal determinants of testicular germ cell tumor by histological subtypes: The EPSAM1 and EPSAM2 studies

**DOI:** 10.1002/ijc.70083

**Published:** 2025-08-13

**Authors:** Mauro Cioffi, Giovenale Moirano, Elena Isaevska, Valentina Fiano, Massimo Di Maio, Patrizia Lista, Ilaria Depetris, Andrea Zitella, Pietro Quaglino, Lorenzo Richiardi, Maja Popovic

**Affiliations:** ^1^ Cancer Epidemiology Unit, Department of Medical Sciences University of Turin and CPO Piemonte Turin Italy; ^2^ Division of Medical Oncology 1 U, Department of Oncology A.O.U. “Città della Salute e della Scienza di Torino” Turin Italy; ^3^ Department of Oncology University of Turin Turin Italy; ^4^ Division of Urology, Department of Surgical Sciences A.O.U. “Città della Salute e della Scienza di Torino” Turin Italy; ^5^ Dermatology Clinic, Department of Medical Sciences University of Turin Turin Italy

**Keywords:** non‐seminomas, anthropometrics, case–control, postnatal factors, seminomas, testicular cancer

## Abstract

The EPSAM study is an ongoing case–control study evaluating the postnatal risk factors for testicular germ‐cell tumor (TGCT). We aimed at updating the risk estimates for factors identified in previous EPSAM analyses and studying heterogeneities between seminomas and non‐seminomas. TGCT cases (*N* = 358) and controls (*N* = 459) were recruited in two phases (EPSAM1, population‐based, and EPSAM2, hospital based). We analyzed the associations with TGCT the following characteristics: (i) anthropometrics at different ages; (ii) baldness; (iii) sibship size; (iv) physical activity at age 13 years; and (v) hospitalizations up to age 18 years. Odds Ratios (ORs) for TGCTs and Relative Risk Ratios (RRRs) for seminomas and non‐seminomas were estimated using unconditional and multinomial logistic regression models. We observed an increased risk of TGCT for height in childhood (OR: 1.83, 95% CI: 1.16–2.90, for taller vs. shorter than peers), pubertal height (OR: 1.40, 0.92–2.11, for taller vs. shorter than peers), adult height above the parental genetic target (OR: 1.47; 1.04–2.07), and any hospitalization in the first 18 years, in particular for diseases with an immune pathogenesis (OR 2.93, 1.33–6.44). Baldness, sibship size, and competitive sports at age 13 years were inversely associated with TGCT risk. Although all anthropometric characteristics were associated with seminomas and showed weaker or no associations with non‐seminomas, there was little evidence of heterogeneity between the two, except for adult height compared to target height. The updated results of the EPSAM study support the role of postnatal environmental factors in TGCT etiology, with an overall lack of heterogeneity between seminomas and nonseminomas.

## Graphical abstract

What's new?

While several nongenetic risk factors are linked to testicular germ‐cell tumors (TGCTs), the influence of environmental and hormonal factors remains unclear. This study evaluated the role of postnatal exposures in the etiology of TGCT using data from the ongoing EPSAM case–control investigation in Italy. Analyses show that childhood and adolescent height, as well as exceeding one's genetically predicted height, increase TGCT risk. Associations were observed predominantly for seminomas. The findings reinforce the role of early‐life exposures in testicular carcinogenesis and suggest possibly distinct etiopathogenetic mechanisms for seminomas and nonseminomas, offering new insights for prevention strategies.

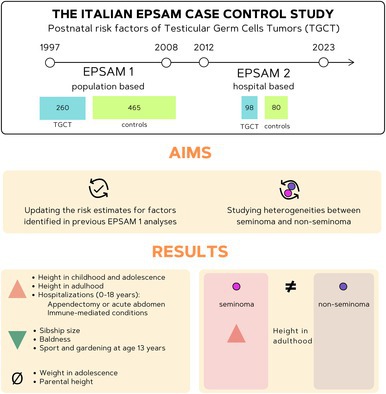

**Abbreviations**
CIs: confidence intervals
DHT: dihydrotestosterone
EPSAM: Esposizioni Postnatali e SAlute Maschile
GCT: germ cell tumor
GP: general practitioner
OR: odds ratio
RRR: relative risk ratio
TGCT: testicular germ cell tumor

## 1 INTRODUCTION

Testicular cancer is a relatively rare neoplasm, accounting for about 1% of malignant tumors in adults. However, it is the most frequent cancer among men aged 15–40 years in many populations, and its incidence is steadily increasing worldwide.^1^

More than 95% of testicular cancers are testicular germ cell tumors (TGCTs), which encompass two main histological subtypes, seminomas and non‐seminomas, with the latter further classified as teratoma, embryonal carcinoma, choriocarcinoma, and yolk sac tumor. Even if TGCT is highly curable, non‐seminomas may have a worse prognosis than seminomas. They also show differences in the germ cell lineage and age of onset of disease. Mixed TGCTs, that is, with both seminomatous and non‐seminomatous components, share the prognosis of pure non‐seminomas.^2^, ^3^

As TCGT occurs in young adults, risk factors for this tumor should act at early age.^4^, ^5^, ^6^ It has been suggested that TCGT originates in utero, but postnatal factors might also play a role, especially exposures acting during puberty, when hormonal changes might further influence germ cells carcinogenesis.^7^

Well‐confirmed non‐genetic risk factors for this neoplasm are cryptorchidism, contralateral testicular cancer, family history of testicular cancer, European origin, and tallness. In the last decades, numerous studies have evaluated environmental and hormonal factors in relation to testicular cancer, usually reporting inconsistent findings.^8^ Moreover, it is not known whether the different histological subtypes might also suggest a difference in risk factors and/or pathogenesis, with the currently available studies providing inconsistent results.^9^, ^10^

In this article, we used data from the EPSAM (*Esposizioni Postnatali e SAlute Maschile*) study, an ongoing Italian case–control study, to estimate the role of several postnatal exposures in the etiology of testicular cancer. The objective was twofold. First, taking advantage of an enlarged sample size (25% increase), we aimed at updating the risk estimates for selected postnatal factors that were found to be associated with TGCT in previous EPSAM analyses,^11^, ^12^, ^13^, ^14^ including (i) height at age 9 and 13 years, adult height, adult height compared to parental height, and weight at age 13 years; (ii) baldness; (iii) sibship size; (iv) physical activity (competitive sport and gardening) at age 13 years; and (v) hospitalizations up to 18 years of age. Second, we aimed at studying heterogeneities in these risk factors between pure seminomas and non‐seminomatous TGCTs.

## 2 MATERIALS AND METHODS

### 2.1 Study design and population

The EPSAM study is a case–control study conducted in the Province of Turin, in the northwest of Italy. The enrollment of participants in the study was conducted in two distinct phases, to which, from now on, we will refer to as “EPSAM1”, population‐based, and “EPSAM2”, hospital‐based.

Full details of the EPSAM1 enrollment have been described in previous publications.^11^, ^12^, ^13^, ^14^ Briefly, EPSAM1 recruitment was carried out using a population‐based approach. Cases were identified through the Regional Discharge Registry among men aged 15–54 years and residents in the Province of Turin who underwent an orchiectomy for testicular cancer between 1997 and 2008. Between 2008 and 2009, the patients who were in follow‐up at the Oncology Ward of the San Giovanni Battista University Hospital (the main hospital of the city of Turin, nowadays named “A.O.U. Città della Salute e della Scienza di Torino”) were contacted through their oncologist; patients who were in follow‐up at other hospitals were contacted through their general practitioners (GPs). Controls (up to two for each case—following a 1:2 case–control design) were selected with the same modalities used to identify the cases: for each case contacted through his GP, we randomly selected two men from the list of the same GP, matched by year of birth (in 5‐year intervals) and residence (city of Turin or rest of the Turin province), and for each case contacted through the Hospital, we contacted up to two patients frequency matched by birth year and residence among patients discharged from the same hospital for non‐neoplastic diseases unrelated to the main known risk factors for testicular cancer. The response rate among subjects contacted through their GPs was 49% for cases and 40% for controls, whereas response rate among subjects contacted through the hospital was 82% among cases and 84% among controls. The study was restricted to TGCTs (14 testicular non germ cell tumors [GCTs] excluded). Two participants (both controls) were excluded from the analyses because of missing information on educational level. Contrary to previous analyses based on the EPSAM1 study,^11^, ^12^, ^13^, ^14^ we included subjects who were not born in Italy (five cases and six controls; but adjusting for this variable in all analyses), resulting in 260 cases and 465 controls.

The EPSAM2 enrollment was carried out using a hospital‐based approach. Cases were identified among patients aged 18–59 years who received a diagnosis of or were followed up for TGCT between 2012 and June 2023 at the urology or oncology wards of the University Hospital “A.O.U. Città della Salute e della Scienza di Torino”. Controls, frequency matched to cases on year of birth (5‐year intervals) following a 1:1 case–control design were selected in the same hospital, among patients of the lithotripsy unit (16%) or the dermatology outpatient clinic (84%) not in treatment for diseases related with the main known risk factors for testicular cancer. The EPSAM2 response rates are 95% and 71% among cases and controls, respectively. Two cases were excluded from the analysis after restriction to GCTs. One control was also excluded because of a previous testicular tumor. Thus, EPSAM2 resulted in an additional 98 cases and 80 controls included in the analysis. The EPSAM2 recruitment is still ongoing.

### 2.2 Exposure assessment

All cases and controls completed a questionnaire that collected information on sociodemographic characteristics (year and place of birth, residence, education level, marital status) and potential risk factors for testicular cancer, focusing mainly on postnatal and pubertal exposures. These included occupations (occasional or professional), anthropometric characteristics at different ages, testicular and reproductive health, physical exercise, hobbies, and other lifestyle habits.

The EPSAM1 questionnaire comprised 94 questions and was sent by mail, with additional information recollected by phone, mail, or e‐mail at the end of the data collection.^11^, ^12^, ^13^, ^14^ For EPSAM2, we developed a shorter version of the same questionnaire (41 questions), which was self‐completed by the study participants at the hospital.

The current analyses focused on five main types of risk factors assessed in the questionnaire: (i) anthropometric characteristics at different ages (height at age 9 and 13 years and in adulthood, weight at age 13 years) and parental height; (ii) baldness; (iii) sibship size; (iv) physical activity (sport and gardening) at age 13 years; and (v) hospitalizations up to 18 years of age.

We selected the aforementioned exposures based on the following criteria: (a) availability of information in both EPSAM1 and EPSAM2 versions of the questionnaire; (b) strength of the association with TGCTs in EPSAM1 findings (i.e. odds ratio ≤0.67 or ≥1.5 and a *p* value ≤0.10 in at least one category of the exposure variables).^11^, ^12^, ^13^, ^14^

In addition, given the distinct age distribution of seminomas and non‐seminomas, we omitted from these analyses age‐dependent exposures (i.e., onset of baldness, number of children born 1 and 5 years before the diagnosis, age at the initial attempt to conceive).

### 2.3 Anthropometric characteristics

The questionnaire recorded information on height in adulthood in cm, height and weight at age 13 years compared with peers, and height when attending elementary school (hereafter referred to as height at age 9 years) compared with peers. Anthropometric characteristics evaluated in comparison with peers were defined in the questionnaire in five levels (“much taller/heavier”, “taller/heavier”, “roughly equal”, “shorter/lighter”, “much shorter/lighter” than peers). We also collected information about the height in cm of the participants' parents.

For statistical analyses, measures at ages 9 and 13 years were grouped into three categories (taller/heavier, same height/weight and shorter/lighter than peers), while adult height was analyzed both as a continuous variable per 5 cm increase and as tertiles according to its distribution among controls.

Similarly, maternal and paternal height were categorized into three categories based on the tertiles of their distribution among controls. Maternal and paternal height were used to estimate the genetic target height using Tanner's formula.^15^ We consequently created a dichotomous variable classifying each subject based on whether their adult height exceeded or did not exceed their target height. Lastly, we evaluated the difference between adult height and the target height, creating a four‐level variable based on the quartiles of the distribution in the control group. These analyses included a smaller number of cases (*N* = 317) and controls (*N* = 411); as in EPSAM1, information about parental height was recollected from study participants at a later stage, using a short, mailed questionnaire.

### 2.4 Baldness, sibship size and physical activity

For baldness, participants were asked to report whether they had ever observed any natural hair loss and, if so, the age at which it started. We created a dichotomic variable considering a lag time to diagnosis of 5 years; thus, subjects who reported starting to lose hair <5 years before the diagnosis for cases and the reference date for controls were considered unexposed. This measure was introduced to avoid reverse causation from the disease and its treatment, but also to try to reduce misclassification bias, because we interviewed cases up to 10 years after the diagnosis, so their recall of the age at which they started to lose hair could be erroneous, in either direction, if the two events (baldness onset and testicular cancer development/treatment) were relatively close in time. Applying a 5‐year time lag led to the reclassification from exposed to unexposed status of 14.3% of cases (*N* = 73), including 12% of pure seminomas (*N* = 23) and 17% of non‐seminomas (*N* = 28), and of 13.5% of controls (*N* = 73). A sensitivity analysis was performed by excluding reclassified individuals from the analyses.

Sibship size was calculated based on the number of siblings reported by each participant. We created a three‐level variable based on the number of children of the parents of each subject, and we also considered it as a continuous variable.

For physical activity, participants were asked to report whether they had practiced competitive sports at the age of 13 years and to specify the type of sport using free text. The questionnaire also asked participants to report on the frequency per month of gardening activities at the age of 13 years, if any. We then created two dichotomous variables, considering whether the study participants had practiced competitive sports or did any gardening activities at age 13 years, respectively.

### 2.5 Hospitalizations up to 18 years of age

Participants reported if they had ever been hospitalized until the age of 18 years and, in case, the cause of each hospitalization (up to nine hospitalizations) as free text.

We created multiple dichotomous variables based on distinct groups of hospitalization causes, not mutually exclusive, analyzing the effect on testicular cancer risk for groups with ≥10 exposed subjects. The definitions of these groups, in part different from the ones used in our previous EPSAM analyses,^14^ are mainly based on previous evidence and/or indication of association with testicular cancer risk in published studies. The complete list of pathologies included in each group can be found in Table S1.

### 2.6 Statistical analyses

The main goals of the statistical analysis were twofold.

First, we estimated odds ratios (ORs) for TGCT, with corresponding 95% confidence intervals (CIs), using unconditional logistic regression for the selected risk factors. In the main text, we report the results of the analyses for the risk factors with an OR ≤0.67 or ≥1.5 and a *p* value ≤0.10 in at least one category. All the other results are reported in the Data S1.

Second, we estimated relative risk ratios (RRRs) with corresponding 95% CIs for the risk factors that were associated with testicular cancer in the first phase of the analysis for pure seminomas and TGCTs with a non‐seminomatous component (i.e., mixed TGCTs or pure non‐seminomas) using multinomial logistic regression. Differences in exposure effects between seminoma and non‐seminomatous TGCTs were tested with the Wald test. Finally, as a sensitivity analysis, we excluded mixed TGCTs, thus comparing pure seminomas with pure non‐seminomas.

We adjusted all models for the matching/study variables, including year of birth (in 5‐year periods), method of contact/identification of the study subjects (EPSAM1‐GPs, EPSAM1‐hospital, EPSAM2) and age at diagnosis (reference date for controls). All models were also adjusted for potential confounders, includingeducational level (primary school, secondary school or college/university degree); cryptorchidism (self‐reported as having been confirmed by a physician, with missing information interpreted as absence of cryptorchidism); country of birth (Italy or rest of the world); and for the analyses on weight at age 13 years, height at the same age (compared to peers). The reference date for the controls was assigned by matching each control to a randomly selected case among those with the closest year of birth to that control.

Analyses were performed using the Software STATA 18 (College Station, TX: StataCorp LP).

## 3 RESULTS

### 3.1 Characteristics of study participants

Overall, 358 cases and 545 controls were included in the analyses, of which 98 cases and 80 controls resulted from the EPSAM2 enrollment (27.4% of cases and 14.7% of controls).

Table 1 summarizes selected characteristics of study participants. Cases and controls were similar in reported characteristics except for cryptorchidism prevalence, which was higher among cases than controls.

**TABLE 1:** Selected characteristics of cases and controls.

characteristic	cases, *n* = 358		controls, *n* = 545	
	*n*	%	*n*	%
*Birth year*				
<1955	19	5.3	47	8.6
1955–1959	29	8.1	56	10.3
1960–1964	35	9.8	70	12.8
1965–1969	49	13.7	79	14.5
1970–1974	63	17.6	102	18.7
1975–1979	69	19.3	96	17.6
1980–1984	34	9.5	38	7.0
1985–1989	28	7.8	31	5.7
1990–1994	16	4.5	10	1.8
1995+	16	4.5	16	2.9
*Recruitment method*				
EPSAM 1 hospital wards/clinics	87	24.3	154	28.3
EPSAM 1 GPs	173	48.3	311	57.1
EPSAM 2	98	27.4	80	14.7
*Birthplace*				
Italy	349	97.5	531	97.4
Other country	9	2.5	14	2.6
*Histology*				
Pure seminomas	193	53.9	–	–
Mixed TGCTs	115	32.1	–	–
Non‐seminomas w.s.c.	50	14.0	–	–
*Educational level*				
Primary school or less	111	31.0	181	33.2
Secondary school	154	43.0	237	43.5
University degree	93	26.0	127	23.3
*Cryptorchidism*				
No	318	88.8	520	95.4
Yes	40	11.2	25	4.6

Abbreviations: GPs, general practitioners; TGCTs, testicular germ cell tumors; w.s.c., without seminomatous component.

The 358 testicular cancer cases were almost equally distributed between pure seminomas (193 subjects, 53.9%) and non‐seminomatous GCTs (165 subjects, 46.1%). The specific histological subtypes of non‐seminoma cases are shown in the Table S2. Non‐seminomatous GCT cases were on average 5 years younger at diagnosis than pure seminoma cases (median age at diagnosis 30.6 vs. 36.1 years). Considering only the 50 non‐seminoma patients without a seminomatous component (30.3% of non‐seminomas and 14.0% of all cases), the age gap increased (median age at diagnosis 29.0 years).

### 3.2 Anthropometric characteristics

Table 2 shows the results for different anthropometric characteristics in all cases, without stratification on histology. Adult height and being taller than peers both at age 13 and 9 years were associated with an increased risk of TGCT, with a more evident association observed for height in childhood compared to adolescence. Although the association between being taller than peers at age 13 and TGCT did not provide evidence against the null hypothesis, the consistent pattern points to a relationship at both age periods. Having reached an adult height higher than the genetic target was also associated with testicular cancer (OR: 1.47; 95% CI: 1.04–2.07%). The difference between adult and target height (whose mean value in controls was +2.50 cm) was positively associated with TGCT risk, with an OR of 1.99 (95% CI: 1.24–3.19) for a difference of at least 6 cm compared to less than −1.5 cm.

**TABLE 2:** Anthropometric characteristics and risk of testicular cancer.

Characteristic	Cases *N* (%)	Controls *N* (%)	OR^a^	95% CI
*Adult height* (*cm*)				
<174	86 (24.2)	171 (31.8)	1.00	Ref
174–178	113 (31.8)	183 (34.1)	1.16	0.81–1.67
179+	156 (43.9)	183 (34.1)	1.61	1.12–2.29
Per 5‐cm increase			1.15	1.03–1.28
*Height age 13 compared to peers*				
Shorter	57 (16.0)	104 (19.2)	1.00	Ref
Same	181 (50.8)	276 (50.9)	1.26	0.86–1.86
Taller	118 (33.2)	162 (29.9)	1.40	0.92–2.11
*p* for linear trend over categories				0.114
*Height age 9 compared to peers*				
Shorter	46 (14.1)	89 (20.9)	1.00	Ref
Same	175 (53.8)	220 (51.9)	1.59	1.04–2.43
Taller	104 (32.0)	115 (27.1)	1.83	1.16–2.90
*p* for linear trend over categories				0.010
*Adult height compared to target height*				
Lower or equal	78 (24.6)	136 (33.1)	1.00	Ref
Higher	239 (75.4)	275 (66.9)	1.47	1.04–2.07
*Difference between adult height and target height, subdivided in quartiles*				
<−1.5 cm	46 (14.5)	86 (20.9)	1.00	Ref
−1.5 to +2.4 cm	76 (24.0)	113 (27.5)	1.27	0.78–2.07
+2.5 to +5.9 cm	75 (23.7)	96 (23.4)	1.49	0.91–2.44
≥6 cm	120 (37.8)	116 (28.2)	1.99	1.24–3.19
*p* for linear trend				0.003

Abbreviations: CI, confidence interval; OR, odds ratio; Ref, reference.

^a^ Odds ratio adjusted for birthplace, birth year (in 5‐year intervals), age at diagnosis, identification/contact method, educational level, and cryptorchidism.

Maternal and paternal height, as well as weight at age 13 years, were not found to be associated with the risk of TGCT in our study (Table S3).

**FIGURE 1:**
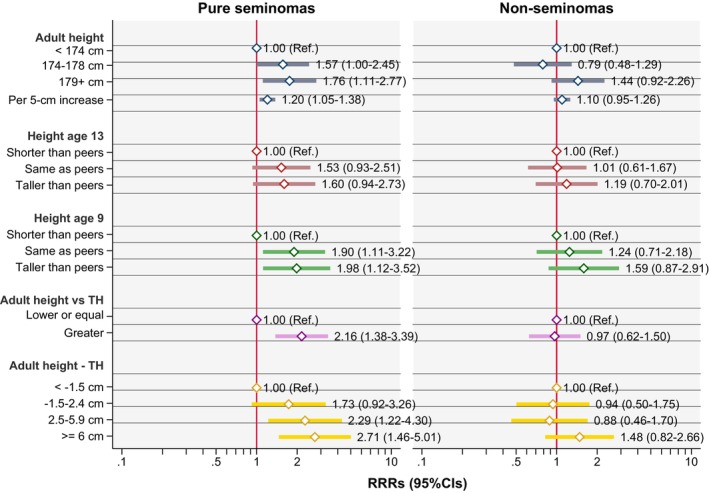
Anthropometric characteristics and risk of testicular cancer: Results for seminomas and non‐seminomatous TGCTs (including mixed TGTCs) vs. controls. RRR adjusted for birthplace, birth year (in 5‐year intervals), age at diagnosis, identification/contact method, educational level, and cryptorchidism. CI, confidence interval; Ref, reference; RRR, relative risk ratio; TGCTs, testicular germ cell tumors; TH, target height.

Figure 1 shows the RRRs of seminomas and non‐seminomatous TGCTs for different anthropometric characteristics. The p‐values of the Wald test for differences in the estimates between seminoma and non‐seminomatous TGCTs are listed in Table S4. All the anthropometric characteristics analyzed showed an association with seminoma, while the relationship with non‐seminomatous TGCTs was generally weaker or absent. In particular, adult height, being taller than peers at age 9, and the difference between adult height and target height were associated with seminoma risk. A similar pattern was also observed for being taller than peers at age 13, though the confidence intervals for both seminomas and non‐seminomatous TGCTs included the null hypothesis. Despite all the anthropometric characteristics showing a clear pattern of association with seminoma only, a Wald test revealed notable heterogeneity only for being taller than one's target height. This characteristic was associated with an increased risk of seminoma (RRR: 2.16, 95% CI: 1.38–3.39) but not with non‐seminomatous TGCTs (RRR: 0.97, 95% CI: 0.62–1.50; Wald test p for heterogeneity = 0.004). The p‐value for heterogeneity in the associations between the two histological subtypes was above 0.05 for all the other anthropometric characteristics analyzed.

The sensitivity analyses restricted to seminomas and non‐seminomas without a seminomatous component yielded comparable results (see Table S5).

### 3.3 Baldness, sibship size, physical activity, and hospitalizations up to 18 years of age

**FIGURE 2:**
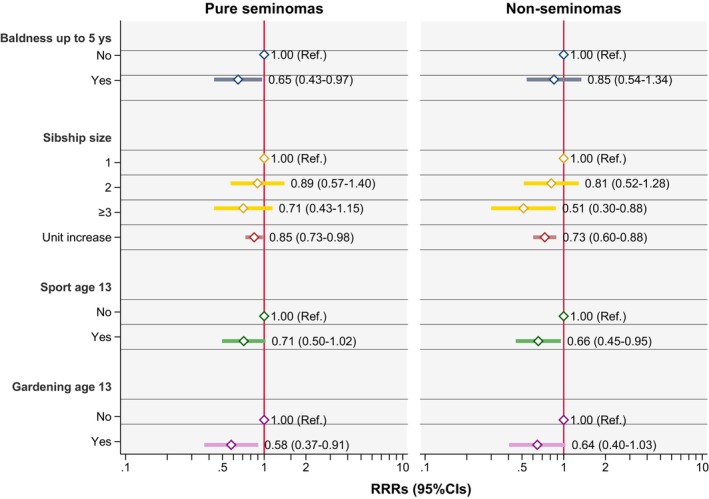
Baldness, sibship size, physical activity, and risk of testicular cancer: Results for seminomas and non‐seminomatous GCTs (including mixed TGTCs) vs. controls. RRR adjusted for birthplace, birth year (in 5‐year intervals), age at diagnosis, identification/contact method, educational level, and cryptorchidism. CI, confidence interval; Ref, reference; RRR, relative risk ratio; TGCTs, testicular germ cell tumors.

Results of the analyses on baldness, sibship size, and physical activity are shown in Table 3. Hair loss starting 5 years before the diagnosis/reference date for controls was associated with a decreased risk of TGCT (OR 0.72, 95% CI: 0.52–1.01). Sibship size was also inversely associated with the risk of TGCT. Finally, both participation in at least one competitive sport and gardening activities at age 13 years were inversely associated with the risk of TGCT, with similar estimates (OR 0.68, 95% CI 0.51–0.91 for sport vs. OR 0.60, 95% CI: 0.42–0.86 for gardening). Figure 2 shows the results of the analysis for seminomas and non‐seminomatous TGCTs separately, while Table S6 reports p‐values of the Wald test for differences in the estimates between seminoma and non‐seminomatous TGCTs. Overall, there was no evidence of substantial heterogeneity in the RRRs of testicular cancer by histological subtype for these exposures (all the estimates are similar between seminoma and non‐seminomatous TGCTs and all p‐values for heterogeneity are above 0.05). Sensitivity analyses restricted to seminomas and non‐seminomatous TGCTs without a seminomatous component provided similar results (see Table S7). The exclusion of individuals reclassified from exposed to unexposed based on a 5‐year time lag for baldness changed the estimates only marginally compared to the main analysis (overall OR = 0.72, 95%CI: 0.50–1.03; pure seminomas RRR = 0.63, 95%CI: 0.41–0.97; non‐seminomas RRR = 0.88, 95%CI: 0.54–1.45).

**TABLE 3:** Baldness, sibship size, physical activity, and hospitalizations and risk of testicular cancer.

Characteristic	Cases *N* (%)	Controls *N* (%)	OR^a^	95% CI
*Baldness arising at least 5 years before the diagnosis of testicular cancer/reference date*				
No	263 (73.5)	367 (67.3)	1.00	Ref
Yes	93 (26.0)	174 (31.9)	0.72	0.52–1.01
*Missing*	2 (0.6)	4 (0.7)		
*Sibship size*				
1	83 (23.2)	107 (19.6)	1.00	Ref
2	181 (50.5)	246 (45.1)	0.86	0.60–1.22
≥3	94 (26.3)	192 (35.2)	0.61	0.41–0.91
Unit increase			0.80	0.71–0.91
*Sport at age 13 years*				
No	205 (57.3)	278 (51.0)	1.00	Ref
Yes	146 (40.8)	257 (47.2)	0.68	0.51–0.91
*Missing*	7 (2.0)	10 (1.8)	–	–
*Gardening at age 13 years*				
No	288 (80.5)	395 (72.5)	1.00	Ref
Yes	61 (17.0)	135 (24.8)	0.60	0.42–0.86
*Missing*	9 (2.5)	15 (2.8)	–	–
*Hospitalizations up to age 18 years*				
No hospitalization	205 (57.3)	357 (65.5)	1.00	Ref
Any hospitalization (excl. cryptorchidism)	142 (39.7)	156 (28.6)	1.51	1.12–2.02
*Missing*	11 (3.1)	32 (5.9)		
*Hospitalization cause* ^b^				
No hospitalization for the specific cause	–	–	1.00	Ref
Genital malformations (excl. cryptorchidism)	7 (2.0)	6 (1.1)	1.64	0.53–5.13
Non genital malformations	8 (2.2)	11 (2.0)	1.12	0.44–2.88
Infections	21 (5.9)	45 (8.3)	0.69	0.40–1.19
Trauma or bone fractures	6 (1.7)	10 (1.8)	0.84	0.30–2.41
Asthma or atopic status	9 (2.5)	5 (0.9)	2.33	0.76–7.22
Tonsillitis/tonsillectomy or adenoiditis/adenoidectomy	47 (13.1)	51 (9.4)	1.48	0.96–2.29
Appendectomy or acute abdomen	33 (9.2)	31 (5.7)	1.74	1.02–2.94
Conditions with immune‐mediated pathogenesis	21 (5.9)	10 (1.8)	2.93	1.33–6.44
Conditions with immune‐mediated pathogenesis (excl. asthma)	12 (3.4)	5 (0.9)	4.01	1.34–11.98
Other conditions	25 (7.0)	30 (5.5)	1.12	0.63–2.00

^a^ Odds ratio adjusted for birthplace, birth year (in 5‐year intervals), age at diagnosis, identification/contact method, educational level, and cryptorchidism.

^b^ Odds ratio calculated only for conditions with ≥10 exposed subjects.

Abbreviations: CI, confidence interval; OR, odds ratio; Ref, reference.

**FIGURE 3:**
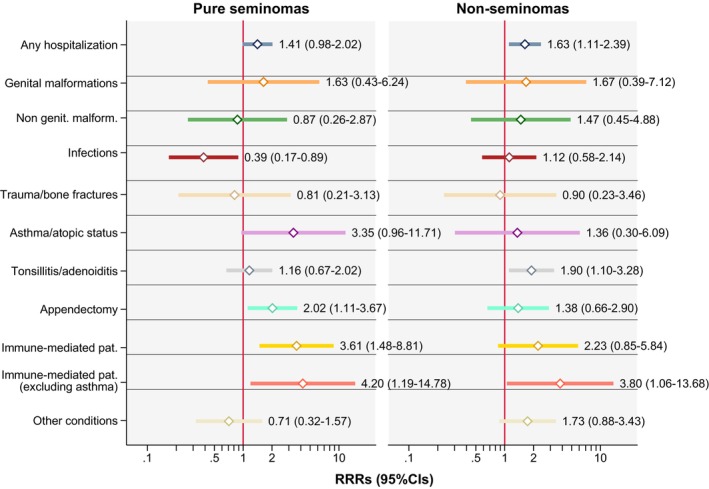
Hospitalizations up to 18 years of age and risk of testicular cancer: Multinomial logistic regression analyses results for seminomas and non‐seminomatous TGCTs (including mixed TGCTs) vs. controls. RRR adjusted for birthplace, birth year (in 5‐year intervals), age at diagnosis, identification/contact method, educational level, and cryptorchidism. RRR calculated only for conditions with ≥10 exposed subjects. CI, confidence interval; Ref, reference; RRR, relative risk ratio; TGCTs, testicular germ cell tumors.

Results on overall and cause‐specific hospitalizations up to age 18 years for all cases, without stratification on histology, are shown in table 3. Results by the two histological subtypes and the test for heterogeneity between the two are shown in Figure 3 and Table S8. The OR for having been hospitalized up to 18 years of age for any cause (excluding cryptorchidism) was associated with testicular cancer (OR: 1.51, 95% CI: 1.12–2.02). This association was similar for seminomas and non‐seminomas. Tonsillitis/tonsillectomy or adenoiditis/adenoidectomy, appendectomy or acute abdomen, and conditions with immune‐mediated pathogenesis were positively associated with the risk of TGCT, but frequently with wide confidence intervals. The strongest positive association was detected for hospitalizations caused by immune‐mediated pathologies (OR 2.93, 95% CI: 1.33–6.44), and the strength of this association increased when we excluded asthma from this exposure (OR 4.01, 95% CI: 1.34–11.98). Among all hospitalization causes, we did not observe any strong heterogeneity in the RRRs of testicular cancer by histological subtype, apart from hospitalizations caused by infections, that were inversely associated only with seminomas (Wald test *p* = 0.031; Figure 3 and Table S8). Sensitivity analyses mostly confirmed these results, but with lower precision (Table S9).

## 4 DISCUSSION

In this case–control study, we evaluated several postnatal exposures in relation to testicular cancer risk and tested potential heterogeneities in the effects of these exposures between pure seminomas and non‐seminomatous TGCTs. The results on all TGCTs, without stratification on histology, update previous findings based on the EPSAM1 data.

Our results suggest that, in addition to adult height, height in childhood/adolescence is positively associated with testicular cancer risk. We also found that exceeding one's target height (calculated based on parental height) is strongly associated with an increased risk of testicular cancer, while parental height itself does not show this positive association. Interestingly, most of these associations were largely or entirely attributed to seminomas. Published studies, including results from previous EPSAM analyses, consistently reported a positive association between adult height and testicular cancer.^16^ However, the evidence associating height in childhood and adolescence (variably measured at age 7 to 13 years) and testicular cancer risk in adulthood is still limited. Only the previous EPSAM findings and a study by McGlynn et al.^17^ evaluated this association, both reporting an increased risk for the tallest subjects in this age interval. It has been hypothesized that height represents a proxy for factors connected to both body growth and testicular cancer. One of these factors could be hormonal status, in particular substances acting as promoters of both statural growth and neoplastic cell proliferation, like sex hormones.^18^ Another hypothesis is that height is correlated to organ size (including the testicle) and the total number of body cells, which are positively associated with cell proliferation and risk of oncogenic mutations.^19^ Also, genetic and/or environmental predisposing factors shared between height and testicular cancer risk could explain their association. Height and testicular cancer could share pleiotropic genetic determinants,^20^ but the evidence supporting this hypothesis is now limited to few genetic variants.^21^, ^22^ Considering parental height as a proxy for the genetic contribution to adult height, our results suggest that the association between height and testicular cancer risk is likely determined by shared environmental rather than genetic factors.

In contrast to the previous findings from the EPSAM study,^14^ in which a lower weight at age 13 years compared to peers was inversely associated with the risk of testicular cancer, this updated analysis did not confirm such an association between weight at age 13 compared to peers and testicular cancer risk. To our knowledge, no other studies have focused specifically on childhood or adolescent weight in relation to testicular cancer risk. Instead, a meta‐analysis of 14 studies, focusing on adult body size, found little evidence of an association between adult weight and BMI with TGCTs. This meta‐analysis reported that overweight (BMI 25–29.9 kg/m^2^) was associated with a slightly reduced risk of TGCT (OR = 0.92; 95% CI: 0.86–0.98), whereas obesity (BMI ≥30 kg/m^2^) showed no clear association with TGCT risk.^23^

In the current study, baldness was inversely associated with testicular cancer, in line with previous EPSAM findings,^12^ even if the strength of the association was reduced. Previous studies mostly support this association.^24^ Baldness can be interpreted as a proxy for the cumulative androgenic stimulation of a subject since puberty, as the pathogenesis of this condition has been linked to dihydrotestosterone (DHT) levels in the hair follicles and the individual sensitivity, genetically determined, to this hormone.^25^ Moreover, androgens showed an inhibitory effect on the growth of seminomatous neoplastic cells both in vitro and in vivo,^26^ and a case–control study found that specific polymorphisms of the androgen receptor gene were associated with testicular cancer risk.^27^

Our finding of sibship size inversely associated with the risk of testicular cancer confirms previous EPSAM results,^13^ but the strength of this association is slightly reduced. Several studies reported an inverse association between the number of siblings and testicular cancer risk.^28^, ^29^, ^30^ However, the meaning of this association is still uncertain, as sibship size could represent a proxy for birth order or an indicator of the genetic predisposition to infertility, which in turn seems to be positively associated with testicular cancer.^31^

In our study, participation in competitive sport and gardening activity during adolescence were inversely associated with the risk of testicular cancer, in line with previous EPSAM results.^14^ A recent systematic review by Huang et al. concluded that the current available evidence is not sufficient to support an association between physical activity and testicular cancer (even if some studies showed a strong protective effect).^32^ As for the potential protective effect of physical activity on testicular cancer risk that we observed, it could be linked to the modifications physiologically induced by exercise, especially in hormonal levels,^33^ but also involving several other aspects, like body composition, immune function, oxidative stress, and epigenetic effects.^34^ To our knowledge, apart from the EPSAM study, gardening activity in relation to testicular cancer risk has not been specifically analyzed by others. While gardening might not be the most common activity for adolescents, it is generally included in structured questionnaires as an example of moderate physical activity^35^ and adolescents may engage in these activities due to family responsibilities, school programs, or personal interest, making it relevant for assessing physical activity. Despite gardening being a proxy for physical activity, it is also a determinant of exposure to pesticides, making its inverse association with testicular cancer somewhat paradoxical. This suggests that the protective effects of physical activity may outweigh potential harmful effects of pesticide exposure. Additionally, adolescents who engage in gardening may have lower overall pesticide exposure (e.g., different gardening practices, organic gardening or limited pesticide use) compared to those in agricultural occupations with higher, more direct pesticide exposure.

Our results on medical history suggest that having been hospitalized at least once for any cause (excluding cryptorchidism) during the first 18 years of life is associated with an increase in testicular cancer risk. Hospitalizations for tonsillitis/tonsillectomy or adenoiditis/adenoidectomy, appendectomy or acute abdomen, and non‐infectious conditions with immune‐mediated pathogenesis (including or excluding asthma in this group) emerged as potential risk factors for testicular cancer. These results partially confirm previous EPSAM findings.^14^ These results must be interpreted cautiously, as they might be to some extent affected by recall bias. However, we found a stronger association with TGCTs for some disease groups than for others, and an inverse association for infectious diseases, suggesting that recall bias does not dominate our findings.

Tonsillectomy has been studied in relation to the development of several cancer types, but the overall evidence on this topic is still conflicting.^36^ Two older studies that examined tonsillectomy and testicular cancer risk found weak evidence of association^37^, ^38^ (Whittermore: RR: 1.4, 95% CI: 0.4–2.7; Swerdlow: OR: 1.07, 95% CI: 0.75–1.52), while a recent large population‐based cohort study reported an increased risk of testicular cancer following surgical removal of tonsils and adenoids (Hazard Ratio: 1.13, 95% CI: 0.98–1.31).^36^ Tonsillectomy might increase cancer risk by impairing the immunologic response and increasing the susceptibility to infection by oncogenic pathogens.^36^ The existing evidence supporting an association between appendectomy and cancer in general is weak, and two studies that assessed specifically testicular cancer did not find any association.^39^, ^40^ The positive association we observed between testicular cancer and conditions with immune‐mediated pathogenesis finds some support in the literature, even if not specifically for this neoplasm, as several studies reported an increased incidence of cancer of any type in subjects affected by autoimmune conditions^41^ and immune‐mediated pathologies in general.^42^ Potential mechanisms include suppression of natural cancer immune surveillance, cellular damage induced by chronic inflammation, and use of immunosuppressive agents.

Concerning the potential heterogeneities of the associations between the selected risk factors and TGCT subtypes, we found evidence of heterogeneity for anthropometric characteristics. The difference in the effect of height on the risk of developing pure seminomas versus non‐seminomas that we found is in line with other studies on matter^16^, ^17^, ^43^, ^44^; however, to our knowledge, the reasons for this finding are not known. The lack of histological heterogeneities for the other selected risk factors, namely baldness, sibship size, physical activity, and hospitalizations up to 18 years of age, is in line with previous studies.^9^, ^10^, ^45^, ^46^

Our study has several potential limitations. First, our results could have been affected by non‐response bias, as in the EPSAM1 recruitment we registered a relatively low participation proportion among cases and controls, particularly for participants contacted through their GPs, while it was higher in the EPSAM2 enrollment. However, because our study focused on exposures that occurred in childhood and adolescence, we think that these are unlikely to be directly associated with participation. Second, we cannot exclude an exposure misclassification due to the time interval spanning between adolescence and the questionnaire. However, it is likely that this misclassification has been non‐differential for most exposures. Third, some of the results of our analysis could have been affected by recall bias. This bias, however, is likely to have affected to some extent only the findings on hospitalizations, as many of the other exposures evaluated are not widely recognized as potential risk factors for testicular cancer (e.g., adult height, number of children). Our results on hospitalizations need further replication, possibly using data from medical records. Finally, it is interesting to note that, like all previous studies, our analysis evaluating the effect of exposures on testicular cancer risk separately for pure seminomas and non‐seminomatous TGCTs was conducted using a prognostic classification to conduct etiological research. This scenario may arise from an incomplete correspondence between the major subtypes of testicular cancer (i.e., seminomas and non‐seminomas) and the underlying pathogenic mechanisms, a fact that could introduce interpretive biases.^46^ A partial confirmation of our results came from the sensitivity analyses, where we observed similar or stronger associations after excluding mixed TGCTs. However, the reduced sample size and subsequent loss of precision often hampered the interpretation and reliability of these findings.

In this study, we conducted hypothesis‐driven analyses, focusing on specific, previously established associations rather than an exploratory search for new ones. Although the issue of multiple testing is relevant, applying corrections such as Bonferroni or Benjamini‐Hochberg may increase Type II errors, potentially masking true associations. Given the prior evidence supporting our hypotheses, we believe that effect sizes, confidence intervals, and consistency with existing research provide a more meaningful framework for interpreting our findings.

In conclusion, our study supports the role of postnatal environmental factors, acting specifically during childhood or adolescence, in the etiopathogenesis of testicular cancer, an aspect that may have strong implications for disease prevention. Some of these factors, in particular those determining growth and final adult height, suggest potentially different etiopathogenetic mechanisms for pure seminomas and non‐seminomatous TGCTs.

## AUTHOR CONTRIBUTIONS

**Mauro Cioffi:** Methodology; data curation; formal analysis; visualization; writing – original draft; investigation. **Giovenale Moirano:** Methodology; data curation; supervision; investigation; writing – review and editing; conceptualization. **Elena Isaevska:** Methodology; data curation; writing – review and editing; investigation. **Valentina Fiano:** Methodology; data curation; resources; writing – review and editing; investigation. **Massimo Di Maio:** Investigation; data curation; writing – review and editing. **Patrizia Lista:** Investigation; data curation; writing – review and editing. **Ilaria Depetris:** Data curation; resources; investigation; writing – review and editing. **Andrea Zitella:** Investigation; writing – review and editing; data curation. **Pietro Quaglino:** Data curation; investigation; writing – review and editing. **Lorenzo Richiardi:** Investigation; conceptualization; methodology; writing – review and editing; supervision; funding acquisition; data curation. **Maja Popovic:** Conceptualization; methodology; data curation; supervision; investigation; writing – original draft; formal analysis; project administration; validation; software.

## FUNDING INFORMATION

This study was partially supported by the Piedmont Region and by the Italian Ministry for Education, University and Research under the program “Dipartimenti di Eccellenza 2018–2022”.

## CONFLICT OF INTEREST STATEMENT

Massimo Di Maio reports honoraria from AstraZeneca, Janssen, Merck Sharp & Dohme (MSD), Novartis, Pfizer, Roche, GlaxoSmithKline, Amgen, Merck, and Takeda for consultancy or participation in advisory boards; direct research funding from Tesaro/GlaxoSmithKline; institutional funding for work in clinical trials/contracted research from Beigene, Exelixis, MSD, Pfizer, and Roche. Other authors report no conflicts of interest.

## ETHICS STATEMENT

The EPSAM study was approved by the local Ethical Committee (EC) of the “AOU Città della Salute e della Scienza” hospital (protocol number 0051727). All study participants provided informed consent by signing a consent form prior to their inclusion in the research.

## Supporting information


**DATA S1.** Supporting information.

## Data Availability

The data that support the findings of this study are available on request from the corresponding author.
