# Supplementary material for: Postnatal determinants of testicular germ cell tumor by histological subtypes: The EPSAM1 and EPSAM2 studies
Source: Int J Cancer. 2025 Aug 13;158(1):108–19. doi: 10.1002/ijc.70083 (PMC12588555; doi:10.1002/ijc.70083)
Supplement: Supplementary file 1 — DATA S1. Supporting information. [file IJC-158-108-s001.pdf]

Postnatal determinants of testicular germ cell tumor by histological subtypes: the EPSAM1 and EPSAM2 studies

Authors

Mauro Cioffi, Giovenale Moirano, Elena Isaevska, Valentina Fiano, Massimo Di Maio, Patrizia Lista, Ilaria Depetris, Andrea Zitella, Pietro Quaglino, Lorenzo Richiardi, Maja Popovic.

Contents:

Table S1 - List of self-reported pathologies included in the groups of hospitalization causes

Table S2 - Histological subtypes of testicular cancer cases.

Table S3 - Anthropometric characteristics and risk of testicular cancer - additional analyses.

Table S4 - Anthropometric characteristics and risk of testicular cancer: results for seminomas and non-seminomas (including mixed GTCs) vs. controls.

Table S5 - Anthropometric characteristics and risk of testicular cancer: results for seminomas and non-seminomas without seminomatous component vs. controls (sensitivity analyses).

Table S6 – Baldness, sibship size, physical activity and risk of testicular cancer: results for seminomas and non-seminomas (including mixed GTCs) vs. controls.

Table S7 – Baldness, sibship size, physical activity and risk of testicular cancer: results for seminomas and non-seminomas without seminomatous component vs. controls (sensitivity analyses).

Table S8 – Hospitalizations up to 18 years of age and risk of testicular cancer: results for seminomas and non-seminomas (including mixed GTCs) vs controls.

Table S9 – Hospitalizations up to 18 years of age and risk of testicular cancer: results for seminomas and non-seminomas without seminomatous component vs. controls (sensitivity analyses).

Table S1 - List of self-reported pathologies included in the groups of hospitalization causes

Hospitalization causes group	Pathologies included
**Genital malformations (excl. cryptorchidism)**	Inguinal hernia, hypospadias
**Non genital malformations**	Hernia, umbilical hernia, talipes equinovarus (clubfoot), renal hypodysplasia, Meckel's diverticulum, cataract, heart murmur, aortic stenosis, Wolff-Parkinson-White syndrome, cleft palate, developmental dysplasia of the hip, supernumerary kidney
**Infections**	Bronchitis, pneumonia, pleuritis, tuberculosis, brucellosis, hepatitis, febrile seizures, enteritis, gastritis, septic arthritis, mononucleosis, measles, nephritis, mumps, meningitis, osteomyelitis, typhoid fever, paratyphoid fever, poliomyelitis, ocular infection, varicella, rubeola, scarlet fever, toxoplasmosis, streptococcal bacteriemia, common cold, food poisoning
**Trauma or bones fractures**	Axis (epistropheus) fracture, radius fracture, accident, head trauma, T10 fracture, lateral malleolus fracture, tibia fracture, wrist fracture, scaphoid fracture, splenectomy after trauma, pelvis fracture, meniscus removal
**Asthma or atopic status**	Asthma, dust allergy, asthmatic bronchitis, anaphylactic shock
**Tonsillitis/tonsillectomy or adenoiditis/adenoidectomy**	Tonsillitis, tonsillectomy, adenoiditis, adenoidectomy
**Appendectomy or other conditions causing acute abdomen**	Appendicitis, appendectomy, peritonitis, kidney stones, testicular torsion, cholecystitis, cholecystectomy, intussusception
**Conditions with immune-mediated pathogenesis**	Post-infective glomerulonephritis, asthma, dust allergy, glomerulonephritis, nephritis, type 1 diabetes mellitus, ABO hemolytic disease, alopecia, thymic hyperplasia, rheumatic fever, food intolerance, Crohn disease, linear IgA bullous dermatosis, Kawasaki syndrome, nephrotic syndrome, bronchospasm, hemolytic anemia, anaphylactic shock
**Other conditions**	Irritable bowel syndrome, abdominal colic, esophageal achalasia, ketosis, pilonidal disease, personality disorder, hydrocele, jaundice, seborrheic dermatitis, secondary polycythemia, pyloric stenosis, deviated septum, thyroid disease, gastric ulcer, varicose veins, retroperitoneal carcinoma, reflux esophagitis, dental extraction, eye pathology, lymphadenectomy, surgical operation, seizures, spontaneous pneumothorax, testicular calcifications, kidney failure, renal transplant, Post-Transplant Lymphoproliferative Disease (PTLD), phimosis, toes amputation, angioma, circumcision, lymphadenopathy, cholesteatoma

Table S2 - Histological subtypes of testicular cancer cases.

Histological subtype	Seminomas N	Seminomas %	Non-seminomas N	Non-seminomas %	All cases %
Pure seminoma	193	100			53.9
Mixed GCTs			115	69.7	32.1
Pure embryonal carcinoma			35	21.2	9.8
Pure choriocarcinoma			1	0.6	0.3
Pure yolk sac tumor			5	3	1.4
Pure teratoma			9	5.4	2.5

Abbreviations: GCTs, Germ Cell Tumors.

Table S3 - Anthropometric characteristics and risk of testicular cancer - additional analyses.

Characteristic	Cases N (%)	Controls N (%)	OR#	95% CI
**Weight age 13 compared to peers‡**				
Same as peers	185 (51.7)	262 (48.1)	1	Ref
Lower than peers	97 (27.1)	179 (32.8)	0.84	0.60-1.17
Higher than peers	74 (20.7)	99 (18.2)	1.00	0.69-1.45
Missing	2 (0.6)	5 (0.9)		
**Maternal height (cm)**				
< 160	68 (21.1)	85 (20.1)	1	Ref
160-164	108 (33.8)	149 (35.3)	0.93	0.61-1.41
165+	145 (45.0)	188 (44.6)	0.97	0.65-1.45
Per 5-cm increase			0.96	0.85-1.09
**Paternal height (cm)**				
< 170	95 (29.7)	120 (28.8)	1	Ref
170-174	82 (25.6)	138 (33.1)	0.72	0.49-1.08
175+	143 (44.7)	159 (38.1)	1.09	0.75-1.59
Per 5-cm increase			1.05	0.93-1.17

OR adjusted for birthplace, birth year (in 5-year intervals), age at diagnosis, cryptorchidism, identification/contact method, and educational lev-el.

‡ ORs for this exposure adjusted for all the aforementioned factors plus height at age 13 years.

Abbreviations: OR: odds ratio; CI: confidence interval; Ref: reference.

Table S4 - Anthropometric characteristics and risk of testicular cancer: results for seminomas and non-seminomas (including mixed GTCs) vs. controls.

Characteristic	Cases N (%)		Controls N (%)	RRR# (95% CI)		Wald test p
**Adult height (cm)**						
< 174	Seminomas	43 (22.5)	171 (31.8)	Seminomas	1.00 (Ref)	
	Non-seminomas	43 (26.2)		Non-seminomas	1.00 (Ref)	
174-178	Seminomas	72 (37.7)	183 (34.1)	Seminomas	1.57 (1.00-2.45)	0.024
	Non-seminomas	41 (25.0)		Non-seminomas	0.79 (0.48-1.29)	
179+	Seminomas	76 (39.8)	183 (34.1)	Seminomas	1.76 (1.11-2.77)	0.491
	Non-seminomas	80 (48.8)		Non-seminomas	1.44 (0.92-2.26)	
Per 5-cm increase				Seminomas	1.20 (1.05-1.38)	0.282
				Non-seminomas	1.10 (0.95-1.26)	
**Height age 13 compared to peers**						
Shorter	Seminomas	27 (14.1)	104 (19.2)	Seminomas	1.00 (Ref)	
	Non-seminomas	30 (18.2)		Non-seminomas	1.00 (Ref)	
Same	Seminomas	103 (53.9)	276 (50.9)	Seminomas	1.53 (0.93-2.51)	0.194
	Non-seminomas	78 (47.3)		Non-seminomas	1.01 (0.61-1.67)	
Taller	Seminomas	61 (31.9)	162 (29.9)	Seminomas	1.60 (0.94-2.73)	0.373
	Non-seminomas	57 (34.5)		Non-seminomas	1.18 (0.70-2.01)	
**Height age 9 compared to peers**						
Shorter	Seminomas	23 (12.8)	89 (21.0)	Seminomas	1.00 (Ref)	
	Non-seminomas	23 (15.7)		Non-seminomas	1.00 (Ref)	
Same	Seminomas	100 (55.9)	220 (51.9)	Seminomas	1.90 (1.11-3.22)	0.227
	Non-seminomas	75 (51.4)		Non-seminomas	1.24 (0.71-2.18)	
Taller	Seminomas	56 (31.3)	115 (27.1)	Seminomas	1.98 (1.12-3.52)	0.555
	Non-seminomas	48 (32.9)		Non-seminomas	1.59 (0.87-2.91)	

**Adult height compared to target height**						
Lower or equal	Seminomas	33 (19.1)	136 (33.1)	Seminomas	1.00 (Ref)	
	Non-seminomas	45 (31.2)		Non-seminomas	1.00 (Ref)	
Higher	Seminomas	140 (80.9)	275 (66.9)	Seminomas	2.15 (1.37-3.38)	0.004
	Non-seminomas	99 (68.8)		Non-seminomas	0.96 (0.62-1.49)	
**Difference between adult height and target height, subdivided in quartiles**						
< -1.5 cm	Seminomas	19 (11.0)	86 (20.9)	Seminomas	1.00 (Ref)	
	Non-seminomas	27 (18.7)		Non-seminomas	1.00 (Ref)	
-1.5-+2.4 cm	Seminomas	43 (24.9)	113 (27.5)	Seminomas	1.73 (0.92-3.26)	0.124
	Non-seminomas	33 (22.9)		Non-seminomas	0.94 (0.50-1.75)	
+2.5-+5.9 cm	Seminomas	48 (27.7)	96 (23.4)	Seminomas	2.29 (1.22-4.30)	0.021
	Non-seminomas	27 (18.7)		Non-seminomas	0.88 (0.46-1.70)	
≥ +6 cm	Seminomas	63 (36.4)	116 (28.2)	Seminomas	2.71 (1.46-5.01)	0.1
	Non-seminomas	57 (39.6)		Non-seminomas	1.48 (0.82-2.66)	
p for linear trend				Seminomas	0.0009	
				Non-seminomas	0.2653	

RRR adjusted for birthplace, birth year (in 5-year intervals), age at diagnosis, identification/contact method, educational level, and cryptorchidism.

Abbreviations: GCTs: germ cell tumors; RRR: relative risk ratio; CI: confidence interval; Ref: reference.

Table S5 - Anthropometric characteristics and risk of testicular cancer: results for seminomas and non-seminomas without seminomatous component vs. controls (sensitivity analyses).

Characteristic	Cases N (%)		Controls N (%)	RRR# (95% CI)		Wald test p
**Adult height (cm)**						
< 174	Seminomas	43 (22.5)	171 (31.8)	Seminomas	1.00 (Ref)	
	Non-seminomas w.s.c.	23 (24.2)		Non-seminomas w.s.c.	1.00 (Ref)	
174-178	Seminomas	72 (37.7)	183 (34.1)	Seminomas	1.56 (0.99-2.45)	0.034
	Non-seminomas w.s.c.	21 (22.1)		Non-seminomas w.s.c.	0.70 (0.37-1.35)	
179+	Seminomas	76 (39.8)	183 (34.1)	Seminomas	1.75 (1.10-2.77)	0.759
	Non-seminomas w.s.c.	51 (53.7)		Non-seminomas w.s.c.	1.57 (0.90-2.77)	
Per 5-cm increase				Seminomas	1.19 (1.04-1.37)	0.306
				Non-seminomas w.s.c.	1.08 (0.90-1.28)	
**Height age 13 compared to peers**						
Shorter	Seminomas	27 (14.1)	104 (19.2)	Seminomas	1.00 (Ref)	
	Non-seminomas w.s.c.	19 (20.0)		Non-seminomas w.s.c.	1.00 (Ref)	
Same	Seminomas	103 (53.9)	276 (50.9)	Seminomas	1.55 (0.94-2.55)	0.134
	Non-seminomas w.s.c.	46 (48.4)		Non-seminomas w.s.c.	0.89 (0.48-1.64)	
Taller	Seminomas	61 (31.9)	162 (29.9)	Seminomas	1.65 (0.96-2.81)	0.143
	Non-seminomas w.s.c.	30 (31.6)		Non-seminomas w.s.c.	0.92 (0.48-1.78)	
**Height age 9 compared to peers**						
Shorter	Seminomas	23 (12.8)	89 (21.0)	Seminomas	1.00 (Ref)	
	Non-seminomas w.s.c.	13 (15.8)		Non-seminomas w.s.c.	1.00 (Ref)	
Same	Seminomas	100 (55.9)	220 (51.9)	Seminomas	1.89 (1.11-3.22)	0.284
	Non-seminomas w.s.c.	43 (52.4)		Non-seminomas w.s.c.	1.21 (0.60-2.46)	
Taller	Seminomas	56 (31.3)	115 (27.1)	Seminomas	1.99 (1.12-3.55)	0.38
	Non-seminomas w.s.c.	26 (31.7)		Non-seminomas w.s.c.	1.35 (0.63-2.89)	

**Adult height compared to target height**						
Lower or equal	Seminomas	33 (19.1)	136 (33.1)	Seminomas	1.00 (Ref)	
	Non-seminomas w.s.c.	27 (33.3)		Non-seminomas w.s.c.	1.00 (Ref)	
Higher	Seminomas	140 (80.9)	275 (66.9)	Seminomas	2.19 (1.39-3.45)	0.001
	Non-seminomas w.s.c.	54 (67.7)		Non-seminomas w.s.c.	0.75 (0.43-1.30)	
**Difference between adult height and target height, subdivided in quartiles**						
< -1.5 cm	Seminomas	19 (11.0)	86 (20.9)	Seminomas	1.00 (Ref)	
	Non-seminomas w.s.c.	27 (18.7)		Non-seminomas w.s.c.	1.00 (Ref)	
-1.5-+2.4 cm	Seminomas	43 (24.9)	113 (27.5)	Seminomas	1.73 (0.92-3.26)	0.182
	Non-seminomas w.s.c.	33 (22.9)		Non-seminomas w.s.c.	0.92 (0.42-2.02)	
+2.5-+5.9 cm	Seminomas	48 (27.7)	96 (23.4)	Seminomas	2.33 (1.24-4.38)	0.011
	Non-seminomas w.s.c.	27 (18.7)		Non-seminomas w.s.c.	0.63 (0.26-1.52)	
≥ +6 cm	Seminomas	63 (36.4)	116 (28.2)	Seminomas	2.73 (1.47-5.06)	0.122
	Non-seminomas w.s.c.	57 (39.6)		Non-seminomas w.s.c.	1.37 (0.65-2.87)	
p for linear trend				Seminomas	< 0.001	
				Non-seminomas w.s.c.	0.642	

RRR adjusted for birthplace, birth year (in 5-year intervals), age at diagnosis, identification/contact method, educational level, and cryptorchidism.

Abbreviations: w.s.c.: without seminomatous component; RRR: relative risk ratio; CI: confidence interval; Ref: reference.

Table S6 – Baldness, sibship size, physical activity and risk of testicular cancer: results for seminomas and non-seminomas (including mixed GTCs) vs. controls.

Characteristic	Cases N (%)		Controls N (%)	RRR# (95% CI)		Wald test p
**Baldness arising at least 5 years before testicular cancer diagnosis/reference date**						
No	Seminomas	136 (70.5)	367 (67.3)	Seminomas	1.00 (Ref)	
	Non-seminomas	127 (77.0)		Non-seminomas	1.00 (Ref)	
Yes	Seminomas	55 (28.5)	174 (31.9)	Seminomas	0.65 (0.43-0.97)	0.316
	Non-seminomas	38 (23.0)		Non-seminomas	0.85 (0.54-1.34)	
Missing	Seminomas	2 (1.0)	4 (0.7)			
	Non-seminomas	0 (0.0)				
**Sibship size**						
1	Seminomas	43 (22.3)	107 (19.6)	Seminomas	1.00 (Ref)	
	Non-seminomas	40 (24.2)		Non-seminomas	1.00 (Ref)	
2	Seminomas	92 (47.7)	246 (45.1)	Seminomas	0.89 (0.57-1.40)	0.733
	Non-seminomas	89 (53.9)		Non-seminomas	0.81 (0.52-1.28)	
≥ 3	Seminomas	58 (30.0)	192 (35.2)	Seminomas	0.71 (0.43-1.15)	0.325
	Non-seminomas	36 (21.8)		Non-seminomas	0.51 (0.30-0.88)	
Unit increase				Seminomas	0.85 (0.73-0.98)	0.187
				Non-seminomas	0.73 (0.60-0.88)	
**Sport at age 13 years**						
No	Seminomas	112 (58.0)	278 (51.0)	Seminomas	1.00 (Ref)	
	Non-seminomas	93 (56.4)		Non-seminomas	1.00 (Ref)	
Yes	Seminomas	75 (38.8)	257 (47.2)	Seminomas	0.71 (0.50-1.02)	0.723
	Non-seminomas	71 (43.0)		Non-seminomas	0.65 (0.45-0.95)	
Missing	Seminomas	6 (3.1)	10 (1.8)			
	Non-seminomas	1 (0.6)				

**Gardening at age 13 years**						
No	Seminomas	152 (78.8)	395 (72.5)	Seminomas	1.00 (Ref)	
	Non-seminomas	136 (82.4)		Non-seminomas	1.00 (Ref)	
Yes	Seminomas	32 (16.6)	135 (24.8)	Seminomas	0.58 (0.37-0.91)	0.714
	Non-seminomas	29 (17.6)		Non-seminomas	0.64 (0.40-1.03)	
Missing	Seminomas	9 (4.7)	15 (2.7)			
	Non-seminomas	0 (0.0)				

RRR adjusted for birthplace, birth year (in 5-year intervals), age at diagnosis, identification/contact method, educational level, and cryptorchidism.

Abbreviations: RRR: relative risk ratio; CI: confidence interval; Ref: reference.

Table S7 – Baldness, sibship size, physical activity and risk of testicular cancer: r results for seminomas and non-seminomas without seminomatous component vs. controls (sensitivity analyses).

Characteristic	Cases N (%)		Controls N (%)	RRR# (95% CI)		Wald test p
**Baldness arising at least 5 years before testicular cancer diagnosis/reference date**						
No	Seminomas	136 (70.5)	367 (67.3)	Seminomas	1.00 (Ref)	
	Non-seminomas w.s.c.	77 (81.1)		Non-seminomas w.s.c.	1.00 (Ref)	
Yes	Seminomas	55 (28.5)	174 (31.9)	Seminomas	0.66 (0.44-0.99)	0.808
	Non-seminomas w.s.c.	18 (18.9)		Non-seminomas w.s.c.	0.72 (0.39-1.31)	
Missing	Seminomas	2 (1.0)	4 (0.7)			
	Non-seminomas w.s.c.	0 (0.0)				
**Sibship size**						
1	Seminomas	43 (22.3)	107 (19.6)	Seminomas	1.00 (Ref)	
	Non-seminomas w.s.c.	24 (25.3)		Non-seminomas w.s.c.	1.00 (Ref)	
2	Seminomas	92 (47.7)	246 (45.1)	Seminomas	0.88 (0.56-1.38)	0.655
	Non-seminomas w.s.c.	49 (51.6)		Non-seminomas w.s.c.	0.76 (0.43-1.34)	
≥ 3	Seminomas	58 (30.0)	192 (35.2)	Seminomas	0.71 (0.43-1.15)	0.721
	Non-seminomas w.s.c.	22 (23.1)		Non-seminomas w.s.c.	0.62 (0.32-1.19)	
Unit increase				Seminomas	0.85 (0.73-0.98)	0.544
				Non-seminomas w.s.c.	0.78 (0.61-0.99)	
**Sport at age 13 years**						
No	Seminomas	112 (58.0)	278 (51.0)	Seminomas	1.00 (Ref)	
	Non-seminomas w.s.c.	53 (55.8)		Non-seminomas w.s.c.	1.00 (Ref)	
Yes	Seminomas	75 (38.8)	257 (47.2)	Seminomas	0.70 (0.48-1.01)	0.87
	Non-seminomas w.s.c.	41 (43.2)		Non-seminomas w.s.c.	0.67 (0.42-1.07)	
Missing	Seminomas	6 (3.1)	10 (1.8)			
	Non-seminomas w.s.c.	1 (1.0)				

**Gardening at age 13 years**						
No	Seminomas	152 (78.8)	395 (72.5)	Seminomas	1.00 (Ref)	
	Non-seminomas w.s.c.	79 (83.2)		Non-seminomas w.s.c.	1.00 (Ref)	
Yes	Seminomas	32 (16.6)	135 (24.8)	Seminomas	0.57 (0.36-0.90)	0.672
	Non-seminomas w.s.c.	16 (16.8)		Non-seminomas w.s.c.	0.67 (0.37-1.21)	
Missing	Seminomas	9 (4.7)	15 (2.7)			
	Non-seminomas w.s.c.	0 (0.0)				

RRR adjusted for birthplace, birth year (in 5-year intervals), age at diagnosis, identification/contact method, educational level, and cryptorchidism.

Abbreviations: w.s.c.: without seminomatous component; RRR: relative risk ratio; CI: confidence interval; Ref: reference.

Table S8 – Hospitalizations up to 18 years of age and risk of testicular cancer: results for seminomas and non-seminomas (including mixed GTCs) vs controls.

Characteristic	Cases N (%)		Controls N (%)	RRR[#] (95% CI)	
**Hospitalizations up to age 18**					
No hospitalization	Seminomas	112 (58.0)	357 (65.5)	Seminomas	1.00 (Ref)
	Non seminomas	93 (56.4)		Non seminomas	1.00 (Ref)
Any hospitalization (excl. cryptorchidism)	Seminomas	76 (39.4)	156 (28.6)	Seminomas	1.41 (0.98-2.02)
	Non seminomas	66 (40.0)		Non seminomas	1.63 (1.11-2.39)
Missing	Seminomas	5 (2.6)	32 (5.9)		
	Non seminomas	6 (3.6)			
**Conditions determining hospitalization up to age 18[§]**					
No hospitalization for the specific condition	Seminomas			Seminomas	1.00 (Ref)
	Non seminomas			Non seminomas	1.00 (Ref)
Genital malformations (excl. cryptorchidism)	Seminomas	4 (2.1)	6 (1.1)	Seminomas	1.63 (0.43-6.24)
	Non seminomas	3 (1.8)		Non seminomas	1.67 (0.39-7.12)
Non genital malformations	Seminomas	4 (2.1)	11 (2.0)	Seminomas	0.87 (0.26-2.87)
	Non seminomas	4 (2.4)		Non seminomas	1.47 (0.44-4.88)
Infections	Seminomas	7 (3.6)	45 (8.3)	Seminomas	0.39 (0.17-0.89)
	Non seminomas	14 (8.5)		Non seminomas	1.12 (0.58-2.14)
Trauma or bones fractures	Seminomas	3 (1.6)	10 (1.8)	Seminomas	0.81 (0.21-3.13)
	Non seminomas	3 (1.8)		Non seminomas	0.90 (0.23-3.46)
Asthma or atopic status	Seminomas	6 (3.1)	5 (0.9)	Seminomas	3.35 (0.96-11.71)
	Non seminomas	3 (1.8)		Non seminomas	1.36 (0.30-6.09)
Tonsillitis/tonsillectomy or adenoiditis/adenoidectomy	Seminomas	22 (11.4)	51 (9.4)	Seminomas	1.16 (0.67-2.01)
	Non seminomas	25 (15.2)		Non seminomas	1.90 (1.10-3.28)
Appendectomy or other conditions causing acute abdomen	Seminomas	22 (11.4)	31 (5.7)	Seminomas	2.02 (1.11-3.67)
	Non seminomas	11 (6.7)		Non seminomas	1.38 (0.66-2.90)
Conditions with immune-mediated pathogenesis	Seminomas	12 (6.2)	10 (1.8)	Seminomas	3.61 (1.48-8.81)
	Non seminomas	9 (5.5)		Non seminomas	2.23 (0.85-5.84)
Conditions with immune-mediated pathogenesis (excl. asthma)	Seminomas	6 (3.1)	5 (0.9)	Seminomas	4.20 (1.19-14.78)
	Non seminomas	6 (3.6)		Non seminomas	3.80 (1.05-13.70)
Other conditions	Seminomas	9 (4.7)	30 (5.5)	Seminomas	0.71 (0.32-1.57)
	Non seminomas	16 (9.7)		Non seminomas	1.73 (0.88-3.43)

\# RRR adjusted for birthplace, birth year (in 5-year intervals), age at diagnosis, identification/contact method, educational level, and cryptorchidism

§ RRR calculated only for conditions with ≥ 10 exposed subjects.

Abbreviations: RRR: relative risk ratio; CI: confidence interval; Ref: reference.

Table S9 – Hospitalizations up to 18 years of age and risk of testicular cancer:
results for seminomas and non-seminomas without seminomatous component vs. controls (sensitivity analyses).

Characteristic		Cases N (%)	Controls N (%)		RRR# (95% CI)	Wald test p
**Hospitalizations up to age 18**						
No hospitalization	Seminomas	112 (58.0)	357 (65.5)	Seminomas	1.00 (Rif)	
	Non-seminomas w.s.c.	53 (55.8)		Non-seminomas w.s.c.	1.00 (Rif)	
Any hospitalization (excl. cryptorchidism)	Seminomas	76 (39.4)	156 (28.6)	Seminomas	1.40 (0.98-2.01)	0.396
	Non-seminomas w.s.c.	39 (41.0)		Non-seminomas w.s.c.	1.77 (1.10-2.85)	
Missing	Seminomas	5 (2.6)	32 (5.9)			
	Non-seminomas w.s.c.	3 (3.2)				
**Conditions determining hospitalization up to age 18§**						
No hospitalization for the specific condition	Seminomas			Seminomas	1.00 (Rif)	
	Non-seminomas w.s.c.			Non-seminomas w.s.c.	1.00 (Rif)	
Genital malformations (excl. cryptorchidism)	Seminomas	4 (2.1)	6 (1.1)	Seminomas	1.59 (0.41-6.12)	0.505
	Non-seminomas w.s.c.	3 (3.2)		Non-seminomas w.s.c.	2.77 (0.64-11.96)	
Non genital malformations	Seminomas	4 (2.1)	11 (2.0)	Seminomas	0.85 (0.26-2.80)	0.135
	Non-seminomas w.s.c.	4 (4.2)		Non-seminomas w.s.c.	2.68 (0.78-9.26)	
Infections	Seminomas	7 (3.6)	45 (8.3)	Seminomas	0.39 (0.17-0.90)	0.100
	Non-seminomas w.s.c.	7 (7.4)		Non-seminomas w.s.c.	1.00 (0.42-2.36)	
Trauma or bones fractures	Seminomas	3 (1.6)	10 (1.8)	Seminomas	0.86 (0.22-3.32)	0.758
	Non-seminomas w.s.c.	2 (2.1)		Non-seminomas w.s.c.	1.16 (0.23-5.88)	
Asthma or atopic status	Seminomas	6 (3.1)	5 (0.9)	Seminomas	3.48 (0.98-12.32)	0.284
	Non-seminomas w.s.c.	2 (2.1)		Non-seminomas w.s.c.	1.31 (0.23-7.52)	
Tonsillitis/tonsillectomy or adenoiditis/adenoidectomy	Seminomas	22 (11.4)	51 (9.4)	Seminomas	1.16 (0.67-2.03)	0.313
	Non-seminomas w.s.c.	14 (14.7)		Non-seminomas w.s.c.	1.73 (0.89-3.40)	
Appendectomy or other conditions causing acute abdomen	Seminomas	22 (11.4)	31 (5.7)	Seminomas	1.99 (1.09-3.62)	0.254
	Non-seminomas w.s.c.	5 (5.3)		Non-seminomas w.s.c.	1.07 (0.39-2.94)	
	Seminomas	12 (6.2)	10 (1.8)	Seminomas	3.69 (1.50-9.04)	0.655

Conditions with immune-mediated pathogenesis	Non-seminomas w.s.c.	7 (7.4)		Non-seminomas w.s.c.	2.90 (1.03-8.18)	
Conditions with immune-mediated pathogenesis (excl. asthma)	Seminomas	6 (3.1)	5 (0.9)	Seminomas	4.17 (1.17-14.83)	0.996
	Non-seminomas w.s.c.	4 (4.2)		Non-seminomas w.s.c.	4.19 (0.99-17.81)	
Other conditions	Seminomas	9 (4.7)	30 (5.5)	Seminomas	0.70 (0.32-1.56)	0.207
	Non-seminomas w.s.c.	7 (7.4)		Non-seminomas w.s.c.	1.41 (0.58-3.46)	

OR adjusted for birthplace, birth year (in 5-year intervals), age at diagnosis, identification/contact method, educational level, and cryptorchidism.

§ OR calculated only for conditions with ≥ 10 exposed subjects.

Abbreviations: w.s.c.: without seminomatous component; OR: odds ratio; CI: confidence interval; Ref: reference.
